# Replacing Fishmeal with Fermented Wheat Protein Improves Nutrient Digestibility and Intestinal Health in Weaned Piglets

**DOI:** 10.3390/ani15162362

**Published:** 2025-08-12

**Authors:** Nuo Xiao, Xiaokang Zhang, Yan Lin, Yuanseng Yang, Yu Wei, Lu Wang, Changhua Lai

**Affiliations:** 1 State Key Laboratory of Animal Nutrition, College of Animal Science and Technology, China Agricultural University, Beijing 100193, China; xiaonuocau@163.com (N.X.); zhangxk0824@163.com (X.Z.); s20223040753@cau.edu.cn (Y.Y.); yuwei20000306@163.com (Y.W.); wanglucau@163.com (L.W.); 2 Hubei Jingruitianheng Biotechechnology Co., Ltd., Yichang 443000, China; lin231405@126.com

**Keywords:** fermented wheat protein, weaned piglets, growth performance, nutrient digestibility, intestinal health

## Abstract

Fermented wheat protein (FWP) has a high protein content and is beneficial to intestinal health, making it an ideal alternative to fishmeal (FM). This study evaluated the effects of replacing FM with FWP on growth performance, nutrient digestibility, and gut health in weaned piglets. A total of 144 weaned piglets (28 days old) were randomly divided into three dietary treatments with 48 piglets per treatment for a duration of 28 days. The treatments included a control diet containing 3% FM (CON group), a diet with 1.5% FM and 1.5% FWP (50% FWP replacement), and a diet with 3% FWP (100% FWP replacement). The results showed that replacing FM with FWP did not significantly affect average daily gain, average daily feed intake, or the feed conversion ratio but significantly reduced diarrhea rates (*p* < 0.01). The 50% and 100% FWP replacement significantly improved the digestibility of crude protein and acid detergent fiber, respectively (*p* < 0.05). The 100% FWP replacement significantly improved the activity of trypsin and chymotrypsin in the duodenum and jejunum (*p* < 0.05). Additionally, the 100% FWP replacement significantly enhanced serum total antioxidant capacity, glutathione peroxidase, and immunoglobulin A contents (*p* < 0.05). The 100% FWP replacement significantly increased villus height in the jejunum (*p* < 0.01). Moreover, the 100% FWP replacement significantly increased the expression of intestinal-barrier-related genes Zonula Occludens-1 and occludin (*p* < 0.01) and decreased the expression of inflammation-related genes (*p* < 0.01). Overall, FWP effectively reduces diarrhea rates, improves gut structure, enhances digestion, and boosts antioxidant and immune functions, particularly at a high replacement ratio (100%). Therefore, FWP can be considered an effective alternative to FM.

## Simple Summary

Piglets are susceptible to a variety of diseases due to weaning stress and the shift in nutrient supply from milk to solid feed, resulting in lower disease resistance. To alleviate weaning stress and prevent a decline in growth rate, nursery pig diets are often supplemented with highly digestible and palatable ingredients. These include animal protein sources such as fishmeal and spray-dried plasma proteins or processed soybean meal products such as soybean protein concentrates and fermented soybean protein concentrates. However, these expensive high-quality proteins increase the cost of pig farming. It is essential to find an efficient and low-cost alternative protein source.

## 1. Introduction

When weaning, piglets are exposed to a variety of stresses, including separation from the sow, transition to solid feed, and changes in feeding conditions. These stresses can lead to digestive disturbances and reduced growth performance in nursery pigs [1,2,3]. To alleviate weaning stress and prevent a decline in growth rate, nursery pig diets are often supplemented with highly digestible and palatable ingredients. These include animal protein sources such as fishmeal (FM) and spray-dried plasma proteins or processed soybean meal products such as soybean protein concentrates and fermented soybean protein concentrates [4,5]. However, these expensive high-quality proteins increase the cost of pig farming. It is essential to find an efficient and low-cost alternative protein source.

Wheat protein (WP) is a major by-product of wheat processing with a protein content 75–80%, which is primarily composed of gluten proteins (gliadin and glutenin). Extensive studies have shown that wheat gluten protein, when properly hydrolyzed, produces small active peptides [6,7]. These active peptides help reduce oxidative stress by removing free radicals and enhancing the activity of the body’s natural antioxidant enzymes. In addition, these peptides can relieve inflammation and protect the intestinal mucosa by lowering inflammatory cytokines and maintaining intestinal barrier integrity [8,9]. Therefore, considering its abundant availability and potential health benefits, fermented wheat protein shows great promise as an alternative to conventional protein feeds. However, wheat protein has several nutritional and functional drawbacks in weaned piglet diets compared to traditional protein sources such as FM, mainly due to its imbalanced amino acid profile, particularly deficiencies in lysine, threonine, methionine, and tryptophan [10,11]. Furthermore, wheat protein contains multiple anti-nutritional factors (ANFs), including non-starch polysaccharides (NSPs), protease inhibitors, and phytic acid. Each of these can negatively impact nutrient utilization and gut health in weaned piglets [12]. Moreover, gluten itself is difficult for young piglets to digest fully. Undigested gluten can reach the hindgut, causing irritation, inflammation, diarrhea, reduced feed intake, slower growth, and lower feed efficiency [13].

Fermentation and enzymatic hydrolysis are processing techniques that improve the nutritional quality and suitability of plant proteins for piglets. Fermentation with beneficial microbes or the addition of exogenous enzymes can partially hydrolyze proteins into smaller peptides and free amino acids, effectively pre-digesting the protein [14,15]. Currently, wheat protein processing primarily uses enzymatic hydrolysis. Research indicates that adding hydrolyzed wheat gluten to broiler diets can strengthen intestinal tight junctions and alleviate inflammation caused by pathogenic *E. coli* [16]. Similarly, including hydrolyzed wheat gluten in weaned piglet diets improved immune responses and lowered diarrhea incidence [6]. Feeding fermented liquid wheat-based diets improved growth performance, nutrient digestibility, gut microbiota diversity, and intestinal health in pigs [17]. Fermented wheat bran supplemented with yeast culture was reported to promote pig growth, immune function, and a healthy gut microbial balance [18,19]. Despite these findings, there is a lack of comprehensive studies evaluating fermented wheat protein (FWP) as a direct replacement for FM in piglet diets.

Thus, the aim of this study was to assess the feasibility of FWP as a replacement for FM by observing the growth performance, diarrhea incidence, intestinal digestion, and antioxidant and immune indicators in the sera and intestines of weaned piglets.

## 2. Materials and Methods

The experimental design and procedures used in this study were approved by the animal ethical committee of China Agricultural University (Beijing, China; No. AW50605202-1-04) on 5 June 2025 and comply with the requirements of the Chinese Animal Welfare Guidelines. The experimental pigs were purchased from Chengde Jiuyun Agricultural and Livestock Co., Ltd. (Chengde, China). The experiment was conducted at the FengNing Swine Research Unit of China Agricultural University (Chengde Jiuyun Agricultural and Livestock Co., Ltd., Chengde, China).

### 2.1. Experimental Design and Diets

One hundred and forty-four 28 d old Duroc × (Landrace × Yorkshire) piglets (6.89 ± 0.71 kg) were randomly divided into 3 groups according to a completely randomized design based on body weight (BW) and gender (half male and half female). Each group contained 6 replicates (8 piglets per replicate) and 4 barrows and 4 gilts per replicate. Pigs were assigned to 3 dietary treatments, including a diet containing 3% fishmeal (CON group), a diet containing 1.5% fishmeal and 1.5% FWP (50% FWP replacement group), and a diet containing 3% FWP (100% FWP replacement group). The experimental diets were formulated to provide all nutrients to meet or exceed NRC (2012) [20] nutrient requirements (Table 1a). All diets used in this study were free from antibiotic growth promoters (AGP). The FWP was provided by Hubei Jingruitianheng Biotechechnology Co., Ltd. (Yichang, China). The amino acid composition of the FWP and fish meal is shown in Table 1b.

### 2.2. Feeding Management and Sample Collection

Piglets lived in 18 pens (2 m × 3 m) inside a temperature-controlled room kept between 22 °C and 28 °C. For the 28-day test, they could eat and drink freely, and caretakers checked their stool every day. After a 12 h fast, the pigs were weighed on days 14 and 28 to obtain their body weight (BW) and determine their average daily gain (ADG). Feed offered and refusals were recorded by pen; average daily feed intake (ADFI) was the total feed eaten divided by the number of pig-days, and the feed-to-gain ratio (F/G) was ADFI divided by ADG. Feces were scored as 0 (normal), 1 (pasty), 2 (semi-liquid), or 3 (liquid); any score of 1 or more was counted as diarrhea, and diarrhea incidence was calculated by the following formula [4]:

Diarrhea rate (%) = number of piglets with diarrhea during the trial period/ (total number of pigs × number of trial days) × 100.(1)

Fecal samples were collected in bags from collection trays for 4 consecutive days (from d 24 to 27). Each 100 g sample received 10 mL of 10% sulfuric acid and two drops of toluene and was mixed well and frozen at −20 °C. When the trial ended, feces from the same diet were pooled, dried at 65 °C until the weight stayed the same, and then ground through a 1 mm screen for lab tests. On day 29, after a 12 h fast, blood was taken from the anterior vena cava into a non-anticoagulative tube (Shandong Aosite Medical Devices Co., Ltd., Heze, China); serum was spun at 3500 rpm for 15 min at 4 °C and stored at −20 °C. Afterward, 1 pig per pen (four pens per diet, 12 pigs total) was put down. A mid-jejunum piece was cut out—half was fixed in 10% buffered formalin for slides and the other half was frozen in liquid nitrogen for RNA and qPCR work.

### 2.3. Apparent Total Tract Digestibility

The apparent total tract digestibility (ATTD) of nutrients involved various parameters, including dry matter (DM), crude protein (CP), ether extract (EE), neutral detergent fiber (NDF), and acid detergent fiber (ADF). The methodologies proposed by Van Leeuwen et al. (1996) [21] and Kong and Adeola (2014) [22] were utilized as the foundation for these calculations:

Apparent nutrient digestibility (%) = 100 − [(Cr content in the feed/Cr content in the fecal) × (the content of a nutrient in the fecal/the content of a nutrient in the feed)] × 100.(2)

In the above formula, Cr content in the feed (mg kg^−1^ DM) is the chromium concentration measured in the diet offered and Cr content in the feces (mg kg^−1^ DM) is the chromium concentration measured in the fecal sample.

### 2.4. Serum Parameters

The concentrations of total protein (TP), glucose (GLU), and triglyceride (TG) in serum were determined using a fully automatic biochemistry analyzer (Hitachi High-Tech Corp., Tokyo, Japan), and the kits used in the analyzer were obtained from Maccura Biotechnology Co., Ltd. (Chengdu, China).

The levels of serum total antioxidant capacity (T-AOC), total superoxide dismutase (T-SOD), glutathione peroxidase (GSH-PX), tumor necrosis factor-α (TNF-α), interleukin-6 (IL-6), interleukin-10 (IL-10), and immunoglobulin A (IgA) were determined using commercial assay kits (Nanjing Jiancheng Bioengineering Institute, Nanjing, China), following the manufacturer’s instructions.

### 2.5. Intestinal Morphology

For dehydration and clearing steps, the intestine samples were subjected to a dehydration process involving multiple methanol soaks with varying concentrations. Subsequently, xylene was used to eliminate ethanol from the samples. Segments of the jejunum were embedded in paraffin. Cross-sectional slices, approximately 5 μm in thickness, were obtained from the embedded tissue. The prepared slides were then stained using hematoxylin and eosin to facilitate cellular visualization and examination under a light microscope (Euromex Microscopen B.V., Arnhem, The Netherlands) equipped with ImageFocus Plus V2 software (Euromex Microscopen B.V., Arnhem, The Netherlands) at 10× magnification. The villus height was measured from the tip of the villi to the villous–crypt junction, while crypt depth was determined from this junction to the base of the crypt. Measurement data were obtained from 12 complete villi and crypts in three randomly selected regions of each sample.

### 2.6. Digestive Enzyme Activities, Antioxidant Capacity, and Immunoglobulins

The levels of T-AOC, T-SOD, GSH-PX, and IgA in the jejunal tissues, as well as the digestive enzyme activity in duodenal and jejunal digestive juices, were determined in intestinal tissues by commercially available assay kits (Nanjing Jiancheng Bioengineering Institute, Nanjing, China), following the manufacturer’s instructions. The following kits and analytical principles were used.

T-AOC (Cat. No. A015-1): ferric reducing ability (FRAP) method, absorbance at 593 nm; T-SOD (Cat. No. A001-3, WST-1 method): inhibition of the WST-1 formazan formation, 450 nm; GSH-PX (Cat. No. A005): coupled reaction monitoring NADPH oxidation, 340 nm; IgA ELISA (Cat. No. H106): sandwich ELISA, 450 nm; trypsin (Cat. No. A080-2-2): BAPNA substrate, p-nitroaniline release measured at 410 nm; chymotrypsin (Cat. No. A080-3-1): SAPNA substrate, p-nitroaniline release at 410 nm; lipase (Cat. No. A054-2-1): colorimetric assay based on glycerol (or p-nitrophenol) generation, 420 nm (per kit manual).

### 2.7. Real-Time PCR

Total RNA was extracted using TRIzol™ Reagent (Invitrogen, Thermo Fisher Scientific, Carlsbad, CA, USA); RNA was quantified on a NanoDrop™ 1000 spectrophotometer (Thermo Fisher Scientific, Waltham, MA, USA); 2 μg RNA was treated with RQ1 RNase-Free DNase (Promega, Madison, WI, USA) prior to reverse transcription. After that, 2 μL of diluted cDNA was used for real-time PCR. β-Actin was used as the reference gene for this study. Table 2 lists the primers selected for analysis.

### 2.8. Statistical Analysis

In the current study, production performance was used as a statistical unit of pen, but the other indices were used as statistical units of individual pigs, and all data obtained were analyzed by using a One-way Analysis of Variance (ANOVA) as a completely randomized design in SAS software, version 9.4 (SAS Institute Inc., Cary, NC, USA). Tukey’s test was used to determine differences between treatments. Variability in the data is expressed as standard error of the mean (SEM). A value of *p* < 0.05 was considered statistically significant, while 0.05 ≤ *p* ≤ 0.10 was regarded as a significant trend.

## 3. Results

### 3.1. Growth Performance and Diarrhea Rate

Table 3 shows that replacing FM with FWP had no significant effect on BW, ADG, ADFI, and F/G. However, replacing FM with 50% and 100% FWP significantly reduced (*p* < 0.01) the diarrhea rate on all stages.

### 3.2. Apparent Total Tract Digestibility

The result of apparent total tract digestibility is shown in Table 4. In the 50% FWP replacement group, the digestibility of CP, NDF, and ADF was significantly higher than that of the CON group (*p* < 0.05), while the EE was significantly lower (*p* < 0.05) compared to that of the CON group. Additionally, the 100% FWP replacement group showed lower (*p* < 0.05) digestibility of EE compared to the CON group.

### 3.3. Digestive Enzyme Activities

The findings in Table 5 show that the 100% FWP replacement group had significantly increased chymotrypsin and trypsin activities in both the duodenum and jejunum, as well as lipase activity in the jejunum, compared with the control diet (*p* < 0.05). In addition, trypsin and lipase activities in the duodenum and jejunum were also higher in the 50% FWP replacement group than those in the CON group (*p* < 0.05).

### 3.4. Serum Biochemical Parameter

Replacing FM with 100% FWP significantly increased serum levels of T-AOC, GSH-PX, IgA, and TP (*p* < 0.05; Table 6). There were no significant differences observed between the 50% and 100% FWP replacement groups for any serum indicators (*p* > 0.05; Table 6).

### 3.5. Jejunal Villus Morphological Structure

**Figure 1:**
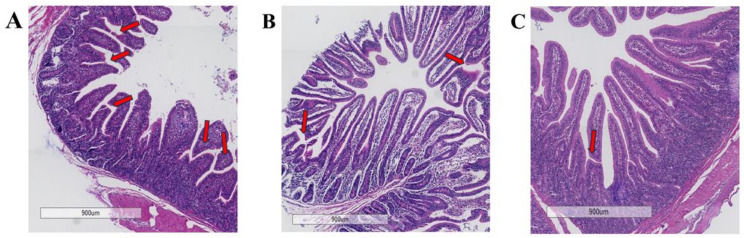
Effects of replacing FM with FWP on jejunum histopathology of weaned piglets: (**A**) a diet containing 3.0% fishmeal; (**B**) a diet containing 1.5% fishmeal and 1.5% FWP FM; (**C**) a diet containing 3.0% FWP. Red arrows indicate sites of villus damage.

Figure 1 depicts severe jejunal villus damage in the CON group, characterized by breakage, atrophy, and detachment. In contrast, replacing FM with 50% and 100% FWP resulted in only slight morphological changes and relatively intact villus structures. Table 7 further demonstrates a significant increase (*p* < 0.05) in jejunal villus height in both FWP replacement groups compared to the FM group.

### 3.6. Jejunal Inflammatory Factors, Immunoglobulins, and Biochemical Indices

Table 8 shows that replacing FM with 50% and 100% FWP significantly increased jejunal GSH-PX content compared to the CON group (*p* < 0.05). However, a significant increase in jejunal IgA content compared to the CON group was observed only when FM was replaced with 100% FWP.

### 3.7. Barrier Function and Inflammatory Cytokines

Table 9 shows that replacing FM with 50% and 100% FWP significantly increased the expression of barrier-function-related genes (*ZO-1* and *Occludin*) compared to the CON group (*p* < 0.05). For inflammatory cytokines (*IL-1α*, *IL-1β*, *IL-6*, and *TNF-α*), significant downregulation was observed only when FM was replaced with 100% FWP compared to the CON group (*p* < 0.05; Table 9).

## 4. Discussion

FM is a widely used protein source in diets for weaned piglets. However, its high price increases pig production costs, creating a need to find suitable alternatives [23]. WP has high yield, but it has a high fiber content, low protein levels, and anti-nutritional factors such as NSP, protease inhibitors, and phytate. Fermentation can pre-digest WP and improve its nutritional value. Therefore, FWP is a potential protein source to replace FM in diets for weaned piglets. Previous studies have found that substitution of FM with high-quality plant protein sources usually does not negatively affect piglet growth if the amino acid balance in the diet is appropriate [24]. This is in agreement with the results of our study. Replacing FM with FWP did not have a negative impact on the growth performance of pigs during d1–14. Similarly, replacing FM with FWP had no significant effect on growth performance on d14–28.

Additionally, we found that FWP increased the digestibility of nutrients, especially the CP, ADF, and NDF. This may be due to the fermentation process acting as a form of pre-digestion [25]. Fermentation produces microbial enzymes such as proteases and cellulases. These enzymes break down complex nutrients into more digestible forms [4,26,27]. Previous studies have similarly reported better digestibility of CP and fiber when piglets were fed fermented soybean or rapeseed proteins due to reduced fiber complexity and anti-nutritional factors [28]. Additionally, we observed that replacing FM with FWP positively improved piglets’ intestinal morphology and increased digestive enzyme activities in the duodenum and jejunum. These changes help enhance nutrient absorption and digestibility. One possible mechanism is that fermentation produces abundant small peptides and amino acids, which can trigger digestive secretions and are absorbed more efficiently in the small intestine [6,29]. For example, several bioactive di-peptides (Leu Tyr, Pro-Tyr, and Tyr-Gln) have been identified in wheat gluten hydrolysates [30]. The increase in serum TP levels observed in the FWP replacement group further indicates improved protein digestion and absorption efficiency. Elevated TP also suggests enhanced hepatic protein synthesis, likely stimulated by improved amino acid availability from fermented peptides [4,24]. In addition, hydrolyzed wheat-gluten peptides have been shown to stimulate hepatic mTOR signaling and albumin synthesis, further supporting the interpretation that elevated TP denotes enhanced whole-body protein anabolism [6].

Weaning stress often leads to oxidative stress, and excessive free radicals can damage the cells of the animal body [31]. Therefore, antioxidant indicators such as T-AOC, GSH-Px, and SOD are important for maintaining the health of weaned piglets. Insufficient antioxidant defenses may increase lipid peroxidation products, harming intestinal and overall health [32,33]. In this study, replacement of FM with FWP significantly increased antioxidant capacity (GSH-PX) in serum and intestinal tissues. In particular, antioxidant enzyme activities were enhanced in the 100% FWP replacement group, indicating an increased ability to scavenge free radicals. Similar results were observed in previous studies, where fermented protein sources increased antioxidant enzyme activity and reduced oxidative stress [34]. The possible reason might be that fermentation produces small peptides with antioxidant properties. These peptides can directly scavenge free radicals and enhance antioxidant systems [35]. For instance, Suetsuna et al. isolated several short peptides (Gly-Tyr and Pro-His-His) with strong antioxidant activity from hydrolyzed wheat gluten, supporting the antioxidant role of fermented products [36]. In addition to antioxidant improvement, higher doses of FWP also positively impacted piglet immunity. This study found that replacement of 100% fishmeal with FWP significantly increased serum and jejunal mucosal immunoglobulin A (IgA) concentrations, indicating that sufficient FWP dosage enhanced humoral immune function [26]. IgA is a primary antibody in mucosal immunity, neutralizing pathogens in the intestine and preventing their adhesion and invasion. Increased IgA levels typically indicate a stronger mucosal immune barrier [37]. Previous studies reported that dietary supplementation with fermented products can improve serum immunoglobulin levels and enhance immune response in weaned piglets [18,19,38]. Likewise, the addition of probiotics or other functional additives has been shown to increase antioxidant and immune markers at relatively high doses [12,37]. Therefore, the significant increase in IgA observed in high-dose FWP supplementation highlights that fermented protein not only provides nutrients but also strengthens antioxidant and immune functions, and these beneficial effects become more prominent with increasing inclusion levels.

Diarrhea is a major problem for piglet production, often causing growth retardation, dehydration, and even death. Therefore, the diarrhea rate is an important indicator for evaluating intestinal health [3]. In this study, replacing FM with FWP significantly reduced diarrhea rates in weaned piglets. The greatest reduction was seen in the 100% FWP replacement group. This positive effect may be due to the fact that fermentation reduces anti-nutritional factors and sensitizing proteins, thereby reducing intestinal stress in piglets [39,40]. Additionally, organic acids produced during fermentation (like lactic and acetic acids) reduce intestinal pH, inhibit harmful bacteria, and support beneficial bacteria, helping maintain gut microbial balance and reducing diarrhea [6,41]. In addition to reducing diarrhea, FWP also positively affected intestinal morphology. Histological observations showed that the villi structure was more intact in the 50% and 100% FWP replacement groups [42,43]. Similar improvements were observed in other studies using fermented feeds or high-quality proteins. Budiño et al. (2005) [42] reported increased jejunal villus height in piglets fed fermented feed combined with prebiotics, and Ma et al. (2019) [4] found improved villus development and reduced diarrhea after replacing FM with enzyme-treated soybean meal. In addition, it has been described above that the replacement of fishmeal with FWP 100% significantly increased the levels of GSH-Px and IgA in the jejunal mucosa, suggesting a reduction in oxidative stress and an increase in mucosal immune function. These changes help protect the intestine from pathogens. At the molecular level, 100% FWP replacement markedly upregulated the mRNA levels of tight-junction proteins (ZO-1 and Occludin) and downregulated pro-inflammatory cytokines (TNF-α and IL-6) in the jejunum, indicating improved intestinal barrier integrity. These effects are likely mediated by bioactive compounds in FWP, such as wheat oligopeptides, which have been shown to enhance tight-junction integrity and alleviate intestinal inflammation through modulation of the TLR4/MyD88/MAPK signaling pathway [8]. Similar findings were reported for other fermented protein feeds. For instance, fermented rapeseed meal improved antioxidant capacity and reduced intestinal inflammation in piglets [34]. Additionally, fermented wheat bran enhanced immune function and reduced inflammatory cytokines in weaned piglets [18,19]. Overall, replacing FM with FWP provided multiple benefits for intestinal health in weaned piglets. The 100% replacement level notably reduced diarrhea, improved intestinal morphology, and strengthened barrier functions, highlighting the advantages of the higher dietary inclusion of fermented protein.

## 5. Conclusions

In conclusion, our results demonstrated that dietary FWP effectively reduced diarrhea incidence and improved intestinal health in weaned piglets by enhancing intestinal morphology, barrier function, and digestive enzyme activities. Specifically, low-level replacement (50%) improved nutrient digestibility, particularly crude protein and fiber components, whereas high-level replacement (100%) significantly increased digestive enzyme activities, antioxidant capacity, and intestinal immune functions. Overall, this study provides clear evidence that FWP, particularly at higher inclusion levels, can serve as an effective alternative protein source to FM in diets for weaned piglets. These findings support the feasibility of incorporating fermented wheat protein into nursery pig nutrition strategies.

## Figures and Tables

**Table 1:** (**a**) Ingredient composition of experimental diets (% as-fed basis). (**b**) Crude protein and amino acid composition of fermented wheat protein versus fishmeal.

(a)			
Items ^1^	CON	50%	100%
Ingredients, %			
Corn	30.73	31.15	31.52
Extruded corn	25.00	25.00	25.00
Wheat flour	6.00	6.00	6.00
Wheat bran	5.00	5.00	5.00
White sugar	2.50	2.50	2.50
Soybean oil	1.00	1.00	1.00
Soybean meal	13.71	13.09	12.42
Full-fat soybean meal	8.00	8.00	8.00
Fishmeal	3.00	1.50	0.00
Fermented wheat protein	0.00	1.50	3.00
Lysine	0.64	0.72	0.80
Methionine	0.21	0.20	0.19
Threonine	0.29	0.30	0.32
L-Valine	0.15	0.15	0.16
Tryptophan	0.10	0.10	0.10
Dicalcium phosphate	0.77	0.93	1.28
Limestone	0.59	0.63	0.55
Salt	0.45	0.45	0.45
Zinc oxide	0.20	0.20	0.20
Choline chloride	0.12	0.12	0.12
Chromium oxide (Cr_2_O_3_)	0.30	0.30	0.30
Premix ^2^	0.50	0.50	0.50
Acidifier	0.30	0.22	0.15
Montmorillonite	0.30	0.30	0.30
Mold inhibitor	0.05	0.05	0.05
Flavoring agent	0.05	0.05	0.05
Antioxidant	0.02	0.02	0.02
Sweetener	0.02	0.02	0.02
Analyzed nutrient levels			
DM ^3^	85.5	85.3	84.3
CP ^4^	25.58	25.44	25.20
EE ^5^	4.46	4.47	4.31
NDF ^6^	10	10	10.1
ADF ^7^	3.63	3.64	3.64
Calculated nutrient levels			
Digestible energy (MJ/kg)	16.45	16.26	16.24
SID ^8^ Lys	1.39	1.38	1.36
SID Met	0.51	0.48	0.46
SID Thr	0.92	0.91	0.91
SID Trp	0.29	0.28	0.28
^1^ CON: a diet containing 3.0% fishmeal; 50%: a diet containing 1.5% fishmeal and 1.5% FWP; 100%: a diet containing 3.0% FWP. ^2^ Premix provides Vitamin A, 12,000 IU; Vitamin D_3_, 2000 IU; Vitamin E, 30 IU; Vitamin K_3_, 3.0 mg; Vitamin B_6_, 3.0 mg; Vitamin B_12_, 12 μg; Riboflavin, 4.0 mg; Thiamine, 1.5 mg; Niacin, 40 mg; Pantothenic Acid, 15 mg; Folic Acid, 0.7 mg; Biotin, 44 μg; choline chloride, 400 mg; copper, 10 mg; iron, 90 mg; zinc, 80 mg; manganese, 30 mg; iodine, 0.35 mg; selenium, 0.3 mg; ^3^ DM: dry material; ^4^ CP: crude protein; ^5^ EE: ether extract; ^6^ NDF: neutral detergent fiber; ^7^ ADF: acid detergent fiber; ^8^ SID: Standardized Ileal Digestibility.			
(**b**)			
**Items ^1^**	**FM**		**FWP**
Crude protein	84.98		68.91
Aspartic acid	2.56		6.27
Threonine	1.97		2.86
Serine	3.72		2.69
Glutamic acid	29.00		8.75
Proline	10.08		2.79
Glycine	2.73		4.31
Alanine	2.15		4.27
Cystine	1.83		0.58
Valine	3.19		3.43
Methionine	1.26		1.92
Isoleucine	2.94		2.91
Leucine	5.52		4.95
Tyrosine	2.62		2.13
Phenylalanine	4.00		2.68
Histidine	1.67		1.64
Lysine	1.31		5.22
Arginine	2.59		4.51
Tryptophan	0.66		0.71
^1^ FM: Fishmeal; FWP: Fermented wheat protein.			

**Table 2:** Gene primers.

Gene	Primer Pairs Sequence (5′–3′)	Accession Number
*TNF* ^1^*-α*	Forward: GAGCGTTGACTTGGCTGTC	NM_204267
	Reverse: AAGCAACAACCAGCTATGCAC	
*IL* ^2^*-1α*	Forward: CCAAGTGCCACCCCGAATGC	JQ_692172
	Reverse: AGGGGAAGAACCATCCGACTCG	
*IL-1β*	Forward: ACTGGGCATCAAGGGCTA	NM_204524
	Reverse: GGTAGAAGATGAAGCGGGTC	
*IL-6*	Forward: AATGTCGAGGCTGTGCAGATT	NM_214399.1
	Reverse: TGGTGGCTTTGTCTGGATTCT	
*NF-κB* ^3^	Forward: AACCGCTTCCATGTTCCGA	NM_001114281.1
	Reverse: TCCGCGAGTTCGGATTCTC	
*Occludin*	Forward: ATGCATTCTCAGCGAGCGTA	NM_001163647.2
	Reverse: AAGGTACCATAGCCTCGGTC	
*ZO* ^4^*-1*	Forward: GAGGATGGTCACACCGTGGT	XM_021098896.1
	Reverse: GGAGCATGCTGTTTTCTCGG	
*Claudin-1*	Forward: GCCCTGCTTTGCAGCTCCTG	NM_021098896.1
	Reverse: TTTCTGGTTGTTCCGACACG	
*β-actin*	Forward: ATGCATCTAGTCGGACAGCC	XM_003357928.4
	Reverse: GTTTGAGGACGCTGGGATGG	

Note. ^1^ TNF: tumor necrosis factor; ^2^ IL: interleukin; ^3^ NF-κB: nuclear factor kappa B; ^4^ ZO-1: Zonula Occludens-1.

**Table 3:** Effects of replacing FM with FWP on the growth performance and diarrhea rate of weaned piglets.

Items ^1^	CON	50%	100%	SEM	*p*-Value
D1 to 14					
D1 BW ^2^, kg	7.92	7.86	7.90	0.75	1.00
ADG ^3^, g	286	293	277	33	0.94
ADFI ^4^, g	425	432	418	46	0.98
F/G ^5^	1.44	1.50	1.46	0.07	0.80
DR ^6^, %	5.11 ^a^	1.92 ^b^	2.54 ^b^	0.25	<0.01
D15 to 28					
D14 BW, kg	11.92	11.97	11.79	1.13	0.99
ADG, g	381	402	429	25	0.43
ADFI, g	564	583	590	55	0.78
F/G	1.50	1.43	1.38	0.07	0.51
DR, %	2.39 ^a^	0.79 ^b^	0.69 ^b^	0.4	<0.01
D1 to 28					
D28 BW, kg	17.28	17.76	17.79	1.33	0.96
ADG, g	328	353	353	25	0.83
ADFI, g	473	517	493	49	0.94
F/G	1.45	1.45	1.39	0.05	0.68
DR, %	3.79 ^a^	1.36 ^b^	1.64 ^b^	0.64	<0.01

Note. ^1^ CON: a diet containing 3.0% fishmeal; 50%: a diet containing 1.5% fishmeal and 1.5% FWP; 100%: a diet containing 3.0% FWP; ^2^ BW: body weight; ^3^ ADG: average daily gain; ^4^ ADFI: feed intake; ^5^ F/G: feed-to-gain ratio; ^6^ DR: diarrhea rate. Different superscript letters within a row indicate significant differences at *p* < 0.05.

**Table 4:** Effects of replacing FM with FWP on the apparent total tract digestibility of weaned piglets.

Items ^1^, %	CON	50%	100%	SEM	*p*-Value
CP ^2^	82.78 ^b^	86.88 ^a^	85.57 ^ab^	0.90	0.02
EE ^3^	75.13	75.63	77.72	0.95	0.16
NDF ^4^	37.44	39.18	42.77	2.08	0.22
ADF ^5^	15.29 ^b^	18.96 ^ab^	24.74 ^a^	2.22	0.04

Note. ^1^ CON: a diet containing 3.0% fishmeal; 50%: a diet containing 1.5% fishmeal and 1.5% FWP; 100%: a diet containing 3.0% FWP; ^2^ CP: crude protein; ^3^ EE: ether extract; ^4^ NDF: neutral detergent fiber; ^5^ ADF: acid detergent fiber. Different superscript letters within a row indicate significant differences at *p* < 0.05.

**Table 5:** Effects of replacing FM with FWP on the digestive enzyme activities of weaned piglets.

Items ^1^ (nmoL/min/g)	CON	50%	100%	SEM	*p*-Value
**Duodenum**					
Chymotrypsin	1972 ^b^	2206 ^b^	2917 ^a^	81	<0.01
Trypsin	247 ^c^	362 ^b^	446 ^a^	19	<0.01
Lipase	5779 ^b^	6783 ^a^	6398 ^ab^	171	<0.01
**Jejunum**					
Chymotrypsin	2577 ^b^	2737 ^b^	3262 ^a^	133	0.02
Trypsin	365 ^b^	463 ^a^	526 ^a^	22	<0.01
Lipase	6425 ^b^	8217 ^a^	7880 ^a^	285	<0.01

Note. ^1^ CON: a diet containing 3.0% fishmeal; 50%: a diet containing 1.5% fishmeal and 1.5% FWP; 100%: a diet containing 3.0% FWP. Different superscript letters within a row indicate significant differences at *p* < 0.05.

**Table 6:** Effects of replacing FM with FWP on the serum biochemical parameters of weaned piglets.

Items ^1^ (nmoL/min/g)	CON	50%	100%	SEM	*p*-Value
T-AOC ^2^, μmol Trolox/mL	3.25 ^b^	3.78 ^b^	4.67 ^a^	0.18	<0.01
T-SOD ^3^, U/L	1469.57	1774.54	1848.70	89.43	0.06
GSH-PX ^4^, IU/L	157.59 ^b^	170.19 ^b^	216.84 ^a^	10.99	<0.01
IgA ^5^, μg/mL	32.86 ^b^	34.88 ^b^	38.41 ^a^	0.72	<0.01
TNF-α ^6^, pg/ml	155.52	197.47	201.65	23.25	0.33
IL ^7^-10, ng/L	197.91	160.78	193.72	14.35	0.18
IL- ^6^, ng/L	343.79	463.07	337.07	52.62	0.20
GLU ^8^, mg/mL	0.56	0.63	0.52	0.06	0.44
TG ^9^, μmol/mL	0.98	1.00	0.94	0.04	0.61
TP ^10^, mg/mL	12.19 ^b^	11.81 ^b^	13.04 ^a^	0.26	0.01

Note. ^1^ CON: a diet containing 3.0% fishmeal; 50%: a diet containing 1.5% fishmeal and 1.5% FWP; 100%: a diet containing 3.0% FWP; ^2^ T-AOC: total antioxidant capacity; ^3^ T-SOD: total superoxide dismutase; ^4^ GSH-PX: glutathione peroxidase; ^5^ IgA: immunoglobulin A; ^6^ TNF-α: tumor necrosis factor-α; ^7^ IL: interleukin; ^8^ GLU: glucose; ^9^ TG: triglyceride; ^10^ TP: total protein. Different superscript letters within a row indicate significant differences at *p* < 0.05.

**Table 7:** Effects of replacing FM with FWP on the jejunal villus morphological structure of weaned piglets.

Items ^1^	CON	50%	100%	SEM	*p*-Value
VH ^2^, μm	356.72 ^b^	555.62 ^a^	608.73 ^a^	38.15	<0.01
CD ^3^, μm	314.26	356.83	378.70	21.61	0.19
VH-to-CD ratio	1.16	1.42	1.40	0.17	0.51

Note. ^1^ CON: a diet containing 3.0% fishmeal; 50%: a diet containing 1.5% fishmeal and 1.5% FWP; 100%: a diet containing 3.0% FWP; ^2^ VH: villus height; ^3^ CD: crypt depth. Different superscript letters within a row indicate significant differences at *p* < 0.05.

**Table 8:** Effect of replacing FM with FWP on antioxidant capacity and immunoglobulin levels in jejunal tissues of weaned piglets.

Items ^1^	CON	50%	100%	SEM	*p*-Value
*T-AOC* ^2^, μmol *Trolox*/mL	3.43	3.45	3.52	0.14	0.88
*T-SOD* ^3^, U/L	1272.28	1412.12	1412.63	44.93	0.09
*GSH-PX* ^4^, IU/L	132.56 ^b^	166.66 ^a^	151.60 ^a^	4.09	<0.01
*IgA* ^5^, μg/mL	31.83 ^b^	32.10 ^b^	33.97 ^a^	0.42	0.02

Note. ^1^ CON: a diet containing 3.0% fishmeal; 50%: a diet containing 1.5% fishmeal and 1.5% FWP; 100%: a diet containing 3.0% FWP; ^2^ T-AOC: total antioxidant capacity; ^3^ T-SOD: total superoxide dismutase; ^4^ GSH-PX: glutathione peroxidase; ^5^ IgA: immunoglobulin A. Different superscript letters within a row indicate significant differences at *p* < 0.05.

**Table 9:** Effects of replacing FM with FWP on barrier function and relative expression of inflammation-related genes in jejunal tissues of weaned piglets.

Items ^1^	CON	50%	100%	SEM	*p*-Value
*ZO-1* ^2^	0.15 ^b^	1.50 ^a^	1.59 ^a^	0.14	<0.01
*Occludin*	0.09 ^b^	2.47 ^a^	1.53 ^a^	0.33	<0.01
*Claudin-1*	1.41	1.95	2.10	0.35	0.38
*IL* ^3^*-1α*	1.37 ^a^	1.11 ^ab^	0.21 ^b^	0.21	0.01
*IL-1β*	1.40 ^a^	1.16 ^a^	0.27 ^b^	0.12	<0.01
*IL-6*	1.97 ^a^	1.88 ^a^	0.29 ^b^	0.23	<0.01
*NF-κB* ^4^	0.92	1.53	0.72	0.36	0.33
*TNF* ^5^*-α*	1.38 ^a^	1.40 ^a^	0.18 ^b^	0.18	<0.01

Note. ^1^ CON: a diet containing 3.0% fishmeal; 50%: a diet containing 1.5% fishmeal and 1.5% FWP; 100%: a diet containing 3.0% FWP; ^2^ ZO-1: Zonula Occludens-1; ^3^ IL: interleukin; ^4^ NF-κB: nuclear factor kappa B; ^5^ TNF: tumor necrosis factor. Different superscript letters within a row indicate significant differences at *p* < 0.05.

## Data Availability

Data are available from the corresponding author on reasonable request.
